# Contemporary applications of artificial intelligence and machine learning in echocardiography

**DOI:** 10.1038/s44325-025-00064-8

**Published:** 2025-06-26

**Authors:** Nastaran Raissi-Dehkordi, Negar Raissi-Dehkordi, Bo Xu

**Affiliations:** 1 School of Medicine, Shahid Beheshti University of Medical Sciences, Tehran, Iran; 2 Robert and Suzanne Tomsich Department of Cardiovascular Medicine, Section of Cardiovascular Imaging, Sydell and Arnold Miller Family Heart, Vascular and Thoracic Institute, Cleveland Clinic, Cleveland, OH USA

**Keywords:** Cardiology, Cardiovascular diseases

## Abstract

Artificial intelligence (AI) and machine learning (ML) are reshaping echocardiography by automating image analysis, reducing variability, and enhancing diagnostic accuracy through tasks such as view classification, image segmentation, and outcome prediction. Key applications include left ventricular ejection fraction assessment and improved valvular disease diagnostics. Limitations include challenges with generalizability, interpretability, and integration into diverse clinical settings. This article provides a contemporary review of AI and ML applications in echocardiography.

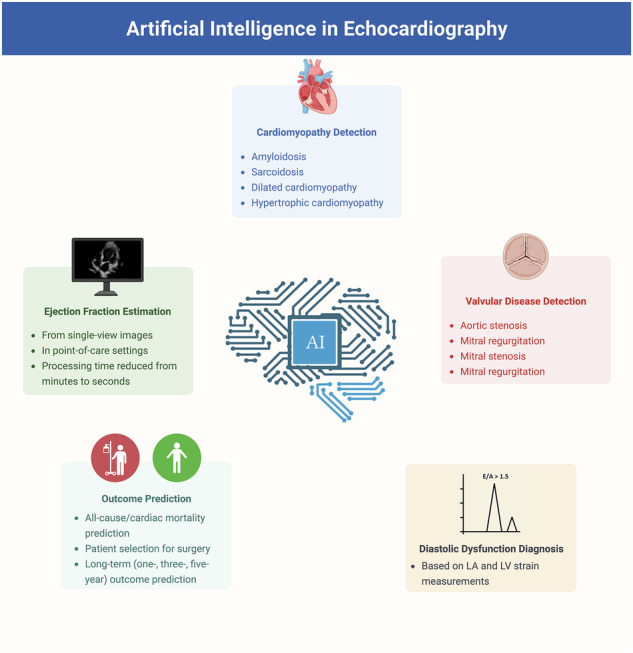

## Introduction

**Fig. 1:**
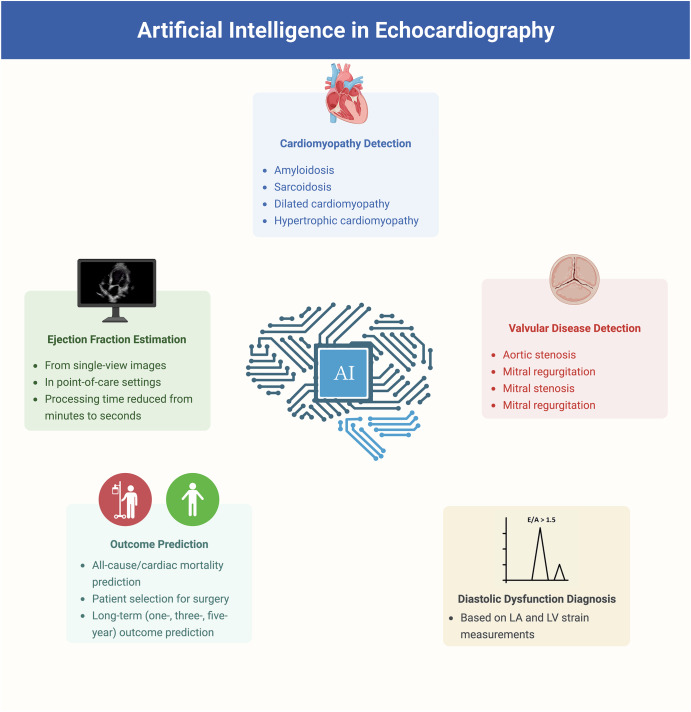
Graphical abstract illustrating the roles of artificial intelligence in echocardiography.

Echocardiography is an integral part of cardiovascular care, enabling non-invasive evaluation of cardiac function across various clinical settings^1^. Despite their utility in evaluating heart failure, diagnosing valvular diseases, and providing point-of-care assessments in emergency situations, current practice of echocardiography faces a number of challenges. Acquisition of ultrasound images by sonography technicians introduces inter- and intra-observer variations^2^, while the laborious and time-consuming nature of interpretation demands expert training^3,4^. Artificial intelligence (AI) has received considerable attention in healthcare research owing to its great potential for clinical application^5^, particularly in medical image analysis^6^, and offers promising solutions for these challenges. Machine learning models efficiently process complex data from echocardiographic images, offering rapid and accurate results. This is crucial given the rich spatiotemporal cardiac image data contained in echocardiographic images, which may be overlooked or inconsistently interpreted by human readers^7^. In this review, we explore the wide range of AI’s applications in echocardiography, including evaluation of cardiomyopathy and assessment of valvular disease (Fig. 1). Additionally, we address current limitations and future directions in integration of AI into routine echocardiography practice.

## Artificial intelligence and machine learning approaches in echocardiography

**Fig. 2:**
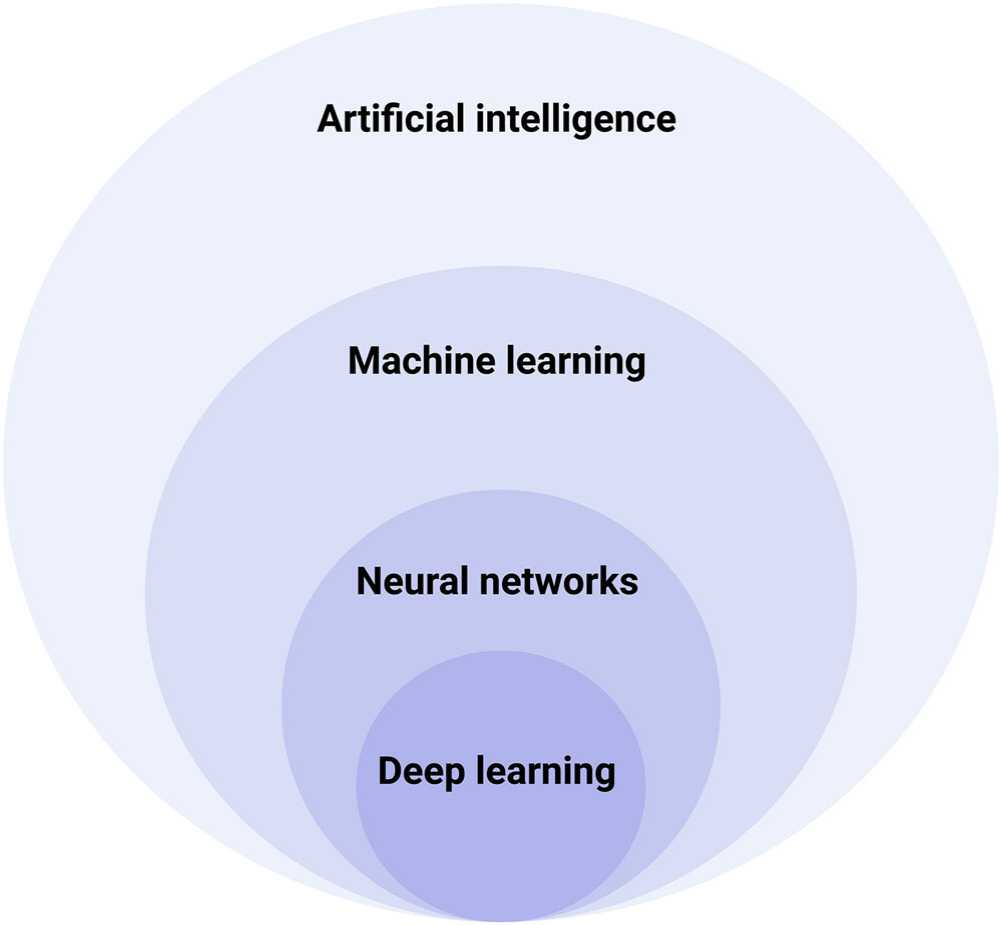
Schematic representation of AI methodologies, including machine learning, deep learning, and neural networks.

AI includes computational techniques that simulate human intelligence, including machine learning (ML), which allows models to learn from data without explicit programming^8^. Deep learning (DL), a more advanced ML approach, uses artificial neural networks to detect complex patterns, especially in image and signal processing, through iterative training that enhances accuracy (Fig. 2). ML methods are typically classified as supervised (e.g., echocardiographic view classification) or unsupervised (e.g., clustering patients based on echocardiographic parameters)^8^. These AI-driven techniques have improved diagnostic consistency and efficiency in echocardiography through applications such as image segmentation, automated quantification, and disease prediction.

For many AI-driven applications, accurate classification of echocardiographic views is a critical first step, ensuring that subsequent analyses are based on correctly identified images. Convolutional Neural Networks (CNNs), which operate like a team of experts detecting patterns at various levels, excel at this task^9^. Each layer of a CNN identifies increasingly complex features—from edges to full shapes—allowing the model to reliably classify images into standard echocardiographic views. However, some deep learning models extract spatial and temporal features directly from echocardiographic data without predefined view classification, allowing for more flexible analysis across varying imaging conditions^10^.

Segmentation takes this task a step further by dividing the image into meaningful anatomical regions, such as the left ventricle (LV) and right ventricle (RV). A specialized CNN variant called U-Net is commonly used for this task^11^. U-Net functions as an encoder-decoder, comparable to zooming in and out of an image. Its unique skip connections act like bookmarks, ensuring that essential details identified during zoom-in (encoding) are not lost during zoom-out (decoding)^12^. This approach allows the model to provide precise boundaries for heart structures, improving the accuracy of measurements such as ejection fraction (EF).

**Fig. 3:**
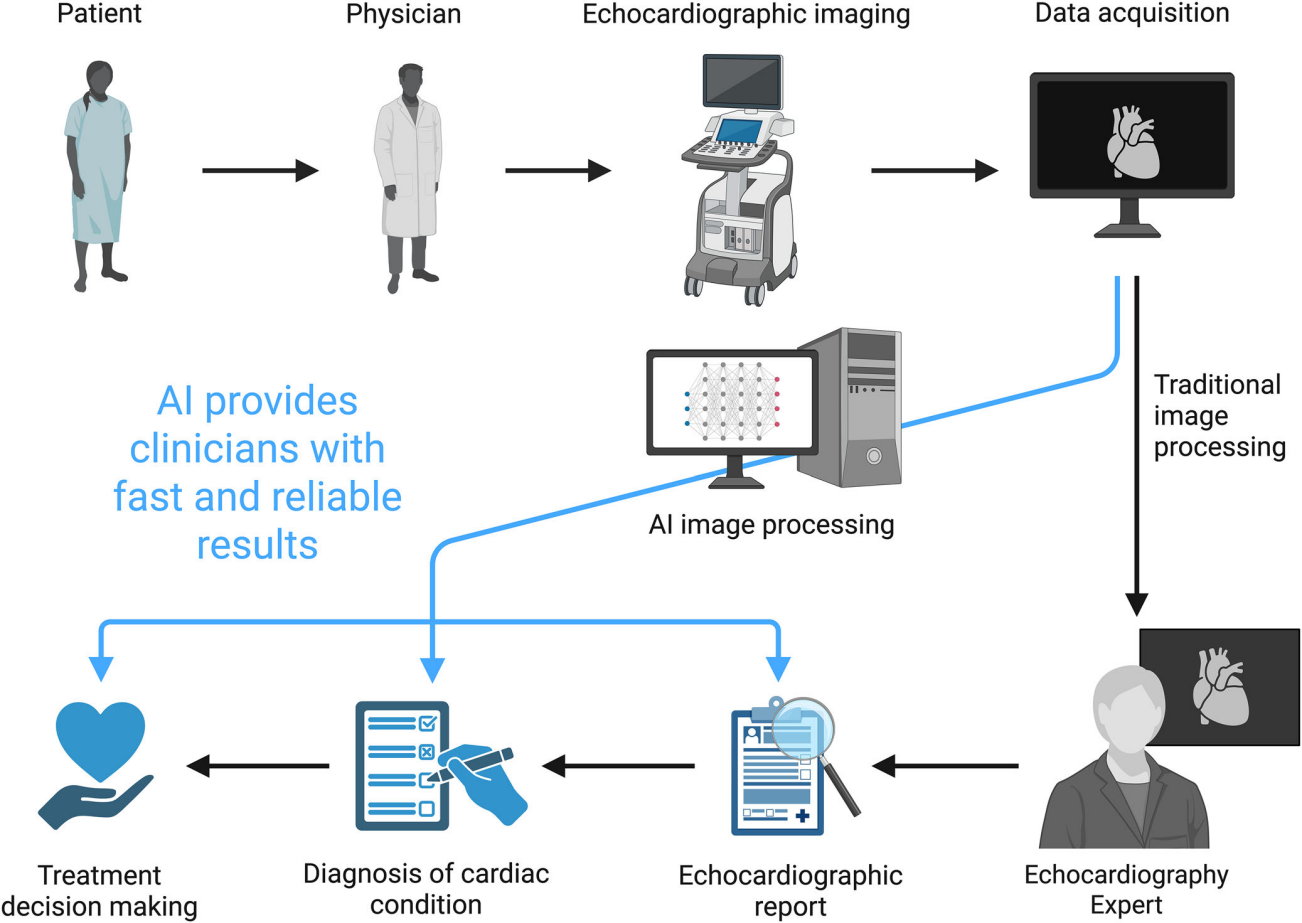
Overview of how artificial intelligence aids in echocardiography.

While deep learning (DL) models perform well at tasks like classification and prediction, they often function as “black boxes,” making it difficult for clinicians to understand how a specific decision was reached. To address this, interpretability techniques such as Gradient-weighted Class Activation Mapping (Grad-CAM) are employed. Grad-CAM works like a highlighter, marking areas of the image that influenced the model’s decision the most^13^. This technique highlights the regions of the image that most strongly influenced the model’s prediction, such as a thickened ventricular wall in hypertrophy. By visualizing what the model is “looking at,” Grad-CAM helps build trust and allows clinicians to assess whether the model’s reasoning aligns with their own interpretation An overview of how AI aids in echocardiography is depicted in Fig. 3.

## Evaluation of ejection fraction

LVEF measurement is the primary measure in assessing systolic function, and reduced LVEF is directly associated with poor cardiac outcomes^14^. LVEF measurement consists of tracking endocardial borders across cardiac cycles, and can be an arduous and time-consuming process^14,15^. ML models have been developed to automate EF measurements, reducing the time needed for image acquisition and analysis while potentially enhancing the accuracy of the results. During echocardiography, multiple views are obtained to achieve a comprehensive understanding of the heart. The initial step for a DL model is to classify these different echocardiographic views^16^. For transthoracic echocardiogram (TTE) images, DL models categorize images into five standard views with high accuracy: the apical two-chamber (A2C), apical four-chamber (A4C), apical three-chamber (A3C), parasternal long axis (PLAX), and parasternal short axis (PSAX)^3^. For transesophageal echocardiograms (TEE), deep learning models have been developed to classify images into eight commonly used views. While this represents a promising first step toward AI-assisted TEE interpretation, clinical applications during intraoperative or intraprocedural use remain to be established^17^.

Once the views are accurately classified, image segmentation is performed to calculate chamber size, identify cardiac structures, and measure wall thickness^18^. A CNN with U-net architecture is commonly used for heart segmentation in two-dimensional (2D) echocardiograms, enabling automatic LVEF measurement based on calculations from three cardiac cycles^19^. By advancing this technique, a spatiotemporal CNN was developed to automatically segment the ventricles and predict EF across frames, helping to create an end-to-end model that performs both segmentation and EF calculation autonomously^20^. An end-to-end DL model estimates EF by analyzing LV volumes across the cardiac cycle, identifying the maximum and minimum ventricular sizes rather than relying on predefined end-systolic and end-diastolic frames. This approach enables direct EF prediction without requiring explicit measurements. The DL model works without predetermined input or guidelines and can infer additional information from the echocardiography image, including identification of intracardiac devices, severe left atrial dilation, and left ventricular hypertrophy, in addition to prediction of age, weight, height, and sex^21^. To further improve accuracy, clinical information could be incorporated into the model, similar to how physicians reach a diagnosis by combining clinical, laboratory, and echocardiographic data.

ML’s versatility allows for the development of models tailored to specific populations by altering the training database. EchoNet-Peds is a DL model developed and trained on over 4000 pediatric echocardiograms, and accurately performs segmentation of LV with a Dice similarity coefficient of 0.89, measures EF with a mean absolute error (MAE) of 3.66% and identifies systolic dysfunction with an area under the curve (AUC) of 0.95 (defined as EF < 55%)^22^.

In addition to EF evaluation, AI is able to assist less experienced physicians and technicians with acquisition of echocardiograms^23^. An innovative study presented a CNN that directly measured LVEF and displayed the estimate on the echocardiography screen to help level I readers improve accuracy, leading to reduced inter-reader variability and more reliable EF measurements across different institutions^23^. Another study provided immediate feedback to the echocardiography operator by addressing image artifacts that might be overlooked during image acquisition, significantly speeding up the process and evaluating more cardiac cycles compared to human measures^24^.

Several ML models have examined LVEF estimation in point-of-care (POC) settings. A group of researchers developed a DL model specifically trained on POC echocardiograms to assist physicians in the emergency department^25^. A dataset of A4C echocardiogram videos obtained in POC settings was annotated for cardiac function and video quality, and EchoNet-POCUS was trained to evaluate both cardiac function and video quality in real-time, achieving an area under the receiver operating characteristic (AUROC) of 0.92 for detecting abnormal EF and an AUROC of 0.81 for assessing video quality^25^. The model by Asch et al. performed reliably well across various views including PLAX or a combination of PLAX and apical views, often matching or exceeding physician performance. The combination of all three views (PLAX, A4C, and A2C) yielded the most accurate results, with no cases exceeding 15% error^26^. The automated model outperformed both cardiologists and POC physicians in classifying LV function using A4C and A2C views. High sensitivity (91.0%) and positive predictive value (PPV) (92.9%) was observed when detecting reduced LV function in the A4C view, with slightly lower but still reliable performance in the A2C and PLAX views^26^. The study analyzed 132 LVEF clips from 44 patients, showing significant agreement between the AI-based LVEF tool and the point-of-care ultrasound (POCUS) expert (Cohen’s Kappa of 0.498, *p* < 0.001), with better results for high-quality clips (Kappa of 0.460). Agreement improved when LVEF was categorized as ≥50% or <50% (Kappa of 0.54, *p* < 0.001)^26^. Similarly, 46 IVC clips showed significant agreement in high-quality clips (intraclass correlation coefficient (ICC) of 0.536, *p* = 0.009)), while 114 left ventricular outflow tract velocity time integral (LVOT VTI) clips demonstrated strong agreement overall (ICC of 0.825, *p* < 0.001)^27^. An ML model applied by Luong et al. showed good agreement with Level III echocardiographers’ estimates of LVEF. The ICC ranged from 0.77 to 0.84, depending on video quality^28^. A summary of studies evaluating AI applications for EF assessment is provided in Table 1.

**Table 1:** AI Models in Ejection Fraction (EF) Assessment

Study	Dataset / Population	Algorithm	Echocardiographic Views	Key Outcomes	AUROC / Accuracy
Ouyang et al.	EchoNet-Dynamic	Spatiotemporal CNN	A4C, A2C, PLAX	Beat-to-beat EF prediction	High agreement
He et al.	POC Emergency Dept	CNN (EchoNet-POCUS)	A4C	EF & video quality	AUROC: 0.92 (EF)
Asch et al.	44 patients, 132 clips	End-to-end DL	A4C, A2C, PLAX	LV function classification	Sensitivity 91%
Luong et al.	132 echo clips	ML ensemble	Various	LV EF prediction	ICC 0.77–0.84

## Evaluation of diastolic dysfunction

Heart failure with preserved ejection fraction (HFpEF) is characterized by signs and symptoms of HF and LVEF ≥ 50%^29^, affecting approximately 6 million individuals in the United States^30^. Despite increasing prevalence, the diagnosis of HFpEF remains a complex and challenging task^31^, and patients often experience recurrent hospitalizations and elevated mortality^32^.

Several ML models have been developed for classification of diastolic dysfunction (DD) and identification of HFpEF. Chen et al. created a system combining view classification, segmentation, and DD grading for assessment of left ventricular diastolic function (LVDF)^33^. The model achieved >90% accuracy using multiview 2D and Doppler data and remained reliable (83–85% accuracy) even when limited to A4C views. Grad-CAM saliency maps confirmed that the model focused on key structures such as the mitral valve, atrial walls, and ventricular septum^33^.

Chiou et al. applied a 1D CNN to A4C data to prescreen for HFpEF, demonstrating strong diagnostic performance (AUC 0.95, accuracy 91%)^34^. The model maintained high accuracy during external validation, identifying subtle changes in LA and LV dynamics^34^. Similarly, Akerman et al. trained a DL model on over 6000 cases using A4C videos, which achieved AUROCs of 0.97 (training) and 0.95 (validation) and reclassified nearly 74% of previously indeterminate HFpEF cases^35^. Grad-CAM was used to provide visual explanations, and confirm that the model focused on relevant cardiac structures such as the left atrium and ventricular walls when making its predictions^35^.

Beyond diagnostic applications, ML can be used to uncover clinically meaningful phenotypes and guide treatment decisions^36^. In the HOMAGE trial, Kobayashi et al. applied an ML model (e′VM algorithm) to stratify high-risk individuals into three subgroups: mostly normal (MN), diastolic changes (D), and diastolic changes with structural remodeling (D/S)^36^. Only patients in the D/S group responded to spironolactone therapy with significant reductions in E/e′ and BNP levels, a phenotype-specific response not captured by conventional heart failure classifications.

Carluccio et al. incorporated peak atrial longitudinal strain (PALS) into an ML algorithm to refine DD classification. The model outperformed traditional guidelines in predicting outcomes (C-index 0.733 vs. 0.720)^37^. Gruca et al. introduced novel parameters like the left atrial strain index (LASi), improving diagnostic clarity in indeterminate cases^38^. At Cleveland Clinic, we conducted a study to assess LVEDP using ML models on data from 460 patients who underwent TTE and cardiac catheterization within 24 h^39,40^. We evaluated multiple ML models, including logistic regression (LR), random forest, gradient boosting, SVM, and K-nearest neighbors (KNN). The LR model demonstrated the highest performance in predicting elevated LVEDP (>20 mmHg), with an AUROC of 0.761. Meanwhile, the gradient boosting model performed best in predicting elevated tau (>45 ms), achieving an AUROC of 0.832. The ML models also classified conditions such as CAD, left ventricular hypertrophy (LVH), and aortic stenosis (AS), with AUROCs ranging from 0.757 to 0.975. Saliency analysis identified key predictors for LVEDP, including E/e′ ratio, NT-proBNP levels, diastolic blood pressure, and left atrial volume^39^. A summary of key studies evaluating ML applications in DD and HFpEF is provided in Table 2.

**Table 2:** AI Models for Diastolic Dysfunction (DD) and HFpEF

Study	AI Approach	Dataset	Echo View	Metrics Used	Accuracy/AUC
Chen et al.	Multiview CNN	TTE + Doppler	A4C + Doppler	LVDF grading	Accuracy >90%
Chiou et al.	1D CNN	1041 HFpEF + controls	A4C	Intrabeat dynamics	AUC 0.95
Akerman et al.	DL classification	2971 HFpEF, 3785 controls	A4C	EF + LA/LV features	AUC 0.95
Carluccio et al.	ML clustering	864 derivation, 189 validation	Multi-view	PALS, E/e′, LAVi	C-index 0.733

## Assessment of right ventricular function

Assessment of RV function is essential in conditions such as pulmonary hypertension, cardiomyopathy, and heart failure with reduced ejection fraction, where impaired RV performance is associated with worse clinical outcomes^41^. However, accurately assessing the RV remains challenging due to its complex geometry, often necessitating the use of multimodal imaging techniques^41^. DL models may be used to address this diagnostic gap. Tokodi et al. developed a DL model using 2D echocardiographic videos to predict right ventricular ejection fraction (RVEF)^42^. The model achieved a MAE of 4.6% (R^2^ = 0.52) on internal validation and 5.5% (R^2^ = 0.45) on external validation. It identified RV dysfunction with high accuracy (AUC 0.93 internal; 0.81 external) and independently predicted major adverse cardiac events (HR 0.924, *p* = 0.025). Saliency maps confirmed attention to the RV myocardium and atrioventricular valves^42^. Their results add to the accumulating evidence on the ability of DL models to offer accurate assessment of RV function and provide prognostic information using standard echocardiographic views. In another example, Kampaktsis et al. used a Transformer-based DL architecture to analyze echocardiographic data, showing high agreement with cardiac MRI in quantifying RV volume and function while reliably detecting RV dysfunction and surpassing conventional 2DE performance (The model achieved an R^2^ of 0.95 for RV volume prediction and an absolute percentage error of 7.2% for RVEF, with 100% detection of RV dysfunction and dilatation)^43^. For predictive applications, Shad et al. trained a video-based DL model using grayscale and optical flow inputs to predict post-operative RV failure in patients undergoing LVAD implantation. The model achieved an AUC of 0.73, outperforming the CRITT score (AUC 0.62), Penn score (AUC 0.61), and clinical cardiologist assessment (AUC 0.58, *p* = 0.016). Saliency maps highlighted key areas such as the RV free wall and atrial borders, though performance declined when predictions were influenced by septal motion^44^. Liu et al. focused on using an AI-based algorithm for RV strain analysis in intraoperative settings^45^. RV-focused views were obtained by TEE and provided vendor-neutral GLS measurements showing strong correlations with conventional metrics. RV fractional area change and GLS demonstrated good agreement (R^2^ = 0.83), while TAPSE correlated with lateral S′ velocity (R^2^ = 0.80)^45^. However, the correlation between GLS and S′ velocity was weaker (R^2^ = 0.40), pointing to differences between longitudinal strain and annular motion measures^45^. AI-based strain analysis could offer a reproducible method for intraoperative RV assessment, though further validation is likely needed. Key studies evaluating AI applications for RV function assessment are summarized in Table 3.

**Table 3:** AI in Right Ventricular (RV) Function Assessment

Study	Algorithm	Dataset	Target Variable	Validation Metrics
Tokodi et al.	CNN	3583 videos	RVEF	AUC: 0.93 (int), 0.81 (ext)
Kampaktsis et al.	Transformer + Feature Tokenizer	50 patients	RV Volumes & RVEF	R^2^ = 0.953
Shad et al.	Two-stream CNN	1909 echos	RV Failure after LVAD	AUC 0.73
Liu et al.	Vendor-neutral AI strain	107 patients	RV GLS	R^2^ = 0.83

## Valvular heart disease evaluation

### Aortic stenosis

Several large-scale studies have demonstrated the utility of DL models trained on 2D echocardiographic videos for AS detection. Holste et al. developed a model using single-view, non-Doppler TTE clips and validated it across multiple external cohorts, achieving AUCs up to 0.98^46^. The model relied on self-supervised learning to extract features efficiently and saliency analysis confirmed its attention to clinically relevant structures such as the aortic valve and mitral annulus^46^. Dai et al. developed a DL model using PLAX-view echocardiographic videos to detect severe AS, defined by guideline-based thresholds for pressure gradient and aortic valve area (AVA)^47^. Trained on over 28,000 studies, the model showed high performance, particularly when using mean pressure gradient as the target, achieving a negative predictive value above 98%. Saliency maps demonstrated that the model focused on the aortic valve and ejection period frames, supporting its use as a screening tool^47^.

In addition to detection, AI has been used to automate the measurement of AS severity parameters such as aortic valve annulus, mean pressure gradient (MPG), and peak velocity (Vmax)^48,49^. Krishna et al. showed strong correlation between automated and human measurements (*r* > 0.88), reinforcing the reliability of AI in quantifying hemodynamic severity^49^. Similarly, automated echocardiographic software has demonstrated strong agreement with CT-based measurements of the aortic annulus, highlighting AI’s potential in valve sizing for TAVR planning^48^.

Wessler et al. developed a model based on PLAX and PSAX views to detect AS across a broader spectrum of severity. The model reached an AUC of 0.96 for any AS and 0.86 for significant AS, with robust external validation performance (AUC 0.91). Saliency maps again confirmed focus on relevant valve structures, suggesting consistent internal logic^50^.

Unsupervised ML techniques, such as hierarchical clustering, can reveal distinct patient subgroups based on echocardiographic and hemodynamic data^51^. Lachmann et al. used hierarchical clustering to uncover four phenogroups among patients undergoing TAVR, each with distinct profiles of cardiac and pulmonary function. Survival varied significantly between clusters, with those showing combined ventricular and pulmonary disease exhibiting substantially lower two-year survival (HR > 2.5)^51^. These clusters highlight how AS affects the cardiovascular system as a whole, providing insights that go beyond traditional linear models^51^. Furthermore, the accuracy of ML models in assessing AS severity can significantly improve decision-making, as they can tailor follow-up schedules based on individual echocardiograms, allowing for a more personalized approach^52,53^.

AI-based tools have also shown promise in tailoring follow-up schedules and identifying high-risk patients missed by traditional criteria^53^. Strange et al. developed an AI decision-support algorithm trained on over one million echocardiograms. The model achieved an AUC of 0.986 in detecting severe AS and flagged individuals with high 5-year mortality (67.9%)—even when they did not meet guideline-defined thresholds. Importantly, outcomes in AI-identified high-risk patients were comparable to those diagnosed through conventional criteria, underscoring AI’s role in earlier detection and improved risk stratification^53^. Table 4 summarizes representative studies on AI-based assessment of aortic stenosis.

**Table 4:** AI in Aortic Stenosis (AS) Evaluation

Study	Dataset	Echo View	Model Type	Key Outcomes	AUROC
Holste et al.	17,570 videos	PLAX	Self-supervised DL	Severe AS detection	0.978
Dai et al.	28,734 studies	PLAX	3 models (MP, MA, MS)	Severe AS + gradient	NPV > 98%
Krishna et al.	3D Echo	Multi-view	DL	Vmax, MPG, AVA	*r* > 0.9
Wessler et al.	8502 scans	PLAX, PSAX	ML (Random Forest)	Detect AS severity	0.96 (any AS)

## Mitral regurgitation and rheumatic heart disease

AI models have been developed to improve mitral regurgitation (MR) risk stratification, grading, and treatment planning. In the EuroSMR study, investigators developed an AI-based risk score incorporating 18 clinical, echocardiographic, laboratory, and medication parameters to better predict 1-year mortality in patients undergoing transcatheter edge-to-edge repair (M-TEER)^54^. The model outperformed conventional risk scores in identifying patients at extreme risk (over 70% mortality) with an AUC of 0.789. The risk score refined patient selection by identifying cases where M-TEER might have been futile. The use of explainable AI techniques such as SHAP highlighted NT-proBNP levels, NYHA class, and TAPSE as key factors influencing the model’s predictions^54^.

While EuroSMR focuses on refining patient selection for surgical intervention, Sadeghpour et al. emphasize precise grading of MR severity^55^. Their multiparametric ML model incorporated 16 parameters, including vena contracta width, regurgitant area ratio, and continuous wave Doppler density, and was trained and validated using two observational cohorts and tested on additional independent data sets, demonstrating 80% accuracy in classifying MR severity from none to severe and 97% accuracy in distinguishing significant MR (moderate or severe) from nonsignificant cases^55^. The approach was robust across both central and eccentric jets, with image analysis feasible in nearly all cases and a mean processing time of 80 s per case^55^. Other models have explored patient subtyping to refine treatment decisions. Bernard et al. developed an ML model using explainable AI to cluster primary MR patients into phenogroups with distinct prognostic outcomes^56^. The model, trained and validated across two cohorts, identified a high-severity group that benefited from mitral valve surgery and a low-severity group that did not show a clear survival advantage^56^. By offering an alternative to conventional severity-grading strategies, AI-based models can help support more personalized management strategies for valvular diseases such as MR.

Several studies have expanded the scope to include other valvular lesions. Yang et al. developed a model trained on more than 2000 studies across institutions to evaluate MR, mitral stenosis (MS), aortic stenosis (AS), and aortic regurgitation (AR)^57^. The model focused on classifying views, detecting lesions, and quantifying key metrics for heart valve diseases (MS AUC 0.99, MR AUC 0.88, AS AUC 0.97, AR AUC 0.90 in the prospective dataset), while also demonstrating comparable performance to expert physicians in identifying key disease metrics, such as MR jet area to left atrial area ratio and peak transvalvular velocity (Vmax) for AS^57^. ML algorithms also enhance phenotyping for conditions such as mitral valve prolapse, improving patient management by identifying subgroups linked to cardiac remodeling and fibrosis^58^. In addition to disease detection and classification, AI has been used to enhance anatomical modeling. Andreassen et al. developed a fully automated deep learning method for mitral annulus segmentation in 3D TEE^59^. The method achieved accurate spatial localization by applying a U-Net architecture and soft-argmax layers, along with temporal regularization to improve continuity across frames. Although limited to systolic frames due to annotation availability, the approach may aid in procedural planning and intraoperative assessment^59^.

AI-based models have also been developed for pediatric echocardiography^60^. Edwards et al. created a two-stage CNN to identify echocardiographic views and detect MR using PLAX recordings. Despite variability in image quality, the system demonstrated robust performance and focused appropriately on critical anatomic structures, as confirmed by CAM^60^. Brown et al. developed a hybrid ML/DL approach to quantify MR jet length in pediatric RHD^61^. Their system closely matched expert manual measurements and achieved high diagnostic performance, particularly in cases with moderate to severe disease^61^.

In the context of rheumatic heart disease (RHD), several studies have leveraged AI to address diagnostic limitations in low-resource settings. Martins et al. developed a video-based 3D CNN system that outperformed traditional 2D approaches, particularly in detecting definite RHD cases^62^. The model was trained on handheld echocardiograms performed by non-experts, reflecting its potential for use in mass screening^62^.

Further addressing the need for diagnostic support in low-resource settings, Peck et al. evaluated an AI-guided approach that allowed novices to capture diagnostic-quality echocardiographic images for RHD screening^63^. The model provided real-time feedback on probe placement and adjustments, enabling novices to capture diagnostic images and achieve diagnostic-quality imaging in over 90% of cases for RHD and mitral valve assessments, though their performance was lower for evaluating aortic valve morphology and stenosis. While experts outperformed novices, novice-acquired scans still resulted in 89% diagnostic agreement with expert evaluations^63^. With minimal training and the help of AI models, non-experts may be able to generate clinically reliable echocardiographic data, opening new possibilities for screening in underserved regions.

Conventional risk scores for MR remain limited, often failing to account for the complex, non-linear cardiopulmonary changes associated with progressive valvular disease. AI models, particularly those using supervised and unsupervised learning, offer an opportunity to capture these dynamic interactions and support more personalized therapeutic decisions^64^. A summary of studies evaluating the role of AI in assessing mitral regurgitation can be found in Table 5.

**Table 5:** AI Models in Mitral Regurgitation (MR) Evaluation

Study	Dataset/Population	Algorithm/AI Method	Application	Key Results	AUROC/Accuracy
Hausleiter et al. (EuroSMR)	4600 patients (4172 derivation, 428 validation)	ML risk score with SHAP explainability	Mortality risk prediction for M-TEER	Identified high-risk patients with >70% mortality	AUC 0.789
Sadeghpour et al.	Multicenter observational cohorts	Multiparametric ML model	MR severity grading	80% accuracy for severity; 97% for moderate/severe MR	Accuracy 80–97%
Bernard et al.	400 patients (France + Canada)	Explainable AI + hierarchical clustering	Phenogrouping for MVS benefit prediction	C-index 0.75; improved reclassification (NRI P = 0.002)	—
Yang et al.	2080 studies from multiple hospitals	CNN + sequence model	Multivalvular disease detection (incl. MR)	MR AUC 0.88; comparable to expert readers	AUC 0.88
Edwards et al.	Pediatric echocardiograms, PLAX views	Two CNNs (view + MR detection)	Pediatric MR detection	High accuracy despite image variability	AUROC 0.91
Martins et al.	11,646 videos from 912 exams	3D CNN + decision tree meta-classifier	RHD diagnosis including MR	Best performance in Definite RHD (85.8%)	Accuracy 72.77%
Peck et al.	36 novices, 50 patients	AI-guided imaging tool	MR image acquisition by novices	90% diagnostic-quality acquisition	—
Brown et al.	511 pediatric echoes	SVM + Transformer + 3D CNN	MR jet length analysis for RHD	Close match with expert grading	AUC 0.93
Trenkwalder et al.	Multicenter MR registry	Unsupervised ML clustering	MR phenotyping for TEER outcome stratification	Identified subgroups with distinct remodeling/fibrosis	—

## Detection of cardiomyopathies

DL models have increasingly been applied to echocardiographic data for the detection and classification of cardiomyopathies. AIEchoDx is an example of a DL framework designed to distinguish among multiple cardiovascular diseases with high precision (AUC 99.50% for ASD, 98.75% for dilated cardiomyopathy (DCM), 99.57% for hypertrophic cardiomyopathy (HCM), and 98.52% for prior MI)^65^. The use of CAM in this model allows for visual interpretation of the model’s decision-making process by localizing specific regions of interest, and shows clinically relevant structures such as the atria in ASD and the interventricular septum in HCM^65^.

Other DL models have been applied to quantify structural features, particularly in HCM and DCM^66^. One model trained on echocardiographic videos from two major centers measured LV wall thickness with a MAE of 1.2 mm, and differentiated HCM from cardiac amyloidosis with AUCs of 0.98 and 0.83, respectively^66^ For cardiac amyloidosis, Goto et al. proposed a two-step AI approach combining ECG and echocardiography^67^. The echocardiography model alone achieved moderate predictive performance (PPV ~ 33%, recall ~67%), but when combined with ECG screening, its PPV improved significantly to over 76% across institutions, with an AUC range of 0.89–1.00^67^. The improved accuracy of combining imaging modalities in AI models becomes especially clear in conditions where diagnosis is more complex, such as cardiac amyloidosis.

AI application has extended to detection of cardiac sarcoidosis (CS), a condition commonly undiagnosed due to its subtle presentations^68^. A DL model trained on A4C echocardiographic views showed limited performance initially (AUC 0.72), but improved to 0.84 after pretraining on a larger dataset (EchoNet-Dynamic)^69^. Interestingly, the model performed better when it was pretrained on a larger dataset and followed by fine-tuning on the smaller CS dataset (sensitivity increased to nearly 90% while specificity remained moderate)^69^. An overview of AI models developed for cardiomyopathy detection is presented in Table 6.

**Table 6:** AI in cardiomyopathy detection

Study	Condition	Algorithm	Dataset	Key Features	Performance
Liu et al. (AIEchoDx)	ASD, DCM, HCM, MI	CAM + DL	Multi-cohort	Disease localization	AUCs > 0.98
Duffy et al.	HCM vs Amyloidosis	DL	Stanford & Cedars-Sinai	Wall thickness	MAE: 1.2–2.4 mm
Goto et al.	Cardiac Amyloidosis	Ensemble DL + ECG	MGH + UCSF	LV strain + ECG	PPV: 76.6%
Katsushika et al.	Cardiac Sarcoidosis	CNN (A4C)	151 CS vs 149 control	Pretraining on EchoNet	AUC 0.84

## Limitations in current artificial intelligence approaches in echocardiography

Despite promising progress, several limitations remain in the path toward widespread clinical integration of AI in echocardiography. The gold standard used to train and evaluate models is typically expert annotations (e.g., estimation of LVEF), which are inherently subjective and prone to inter- and intra-observer variability. When used as training endpoints, they introduce hidden bias into AI models. This creates an illusion of high accuracy when models are simply reproducing the expert’s bias. In other words, an imperfect reference standard may inflate confidence in model results and propagate bias. Study design choices can further exacerbate this problem. The same expert might perform both model training and ground truth comparison, inadvertently favoring intra-observer agreement over inter-observer consistency. This approach gives models an advantage over independent human readers and skews the perception of accuracy.

The limitations of supervised vs unsupervised learning approaches should be considered when choosing and evaluating models. In supervised learning, the model is trained on labeled data, aligning outputs with predefined targets^8^. This allows for transparent and interpretable behavior but restricts the model’s potential to discover novel patterns. Unsupervised learning, by contrast, enables models to find new data structures without human guidance. While promising, these models are harder to interpret and validate—often functioning as black boxes. The “black box” nature of many AI models remains a major barrier to clinical adoption. Without insight into how decisions are made, clinicians may hesitate to trust model outputs—especially when these contradict clinical intuition. Improving model transparency through tools like Grad-CAM and saliency maps is crucial to fostering clinician confidence and enabling safe integration into practice.

Another major challenge is the limited availability of large, annotated databases. Many models are trained on relatively small or institution-specific datasets, increasing the risk of overfitting—where the model memorizes training data instead of learning generalizable features^70^. To create models that perform well across different settings, training datasets must reflect a wide spectrum of patient demographics, disease phenotypes, and imaging quality.

Similarly, models trained on curated datasets from academic centers may not translate well to different populations, particularly in underrepresented or resource-limited settings. This raises the risk of diagnostic errors when models are applied outside their development environment. Broader and more inclusive datasets are key to ensuring reliable performance in varied clinical environments.

Image quality is another significant concern, particularly in POC settings. Handheld devices often produce lower-resolution images with more artifacts, which can degrade model performance if the system was trained on high-quality images. This discrepancy highlights the principle of “garbage in, garbage out”—underscoring the importance of aligning training data with real-world conditions to produce reliable results^58^.

Ultimately, human oversight remains indispensable to the application of AI in cardiac imaging. Physicians must remain the final decision-makers, interpreting AI recommendations within the broader context of clinical findings, patient values, and ethical considerations. AI should be viewed as an assistive tool—not a replacement for human expertise.

## Trends and future directions of AI in echocardiography

Accumulating evidence shows that AI algorithms can achieve diagnostic accuracy comparable to, and in some cases surpassing, clinicians in identifying various cardiovascular pathologies with echocardiography. As these models advance, their diagnostic accuracy is expected to improve with the development of more sophisticated algorithms, and future research is expected to further corroborate these findings through large-scale clinical trials. Nevertheless, the implications of these advancements remain a significant consideration for healthcare providers, patients, and policymakers. While great enthusiasm surrounds AI’s potential applications in clinical imaging and particularly echocardiography, it is essential to maintain cautious optimism when interpreting study findings. The integration of AI into clinical practice requires a thorough understanding of its limitations as mentioned in the section above. Addressing these concerns through ongoing research is essential to ensure the safe and effective use of AI in healthcare.

AI models offer valuable insights and support to physicians, reducing error and improving overall accuracy. Notably, clinicians utilizing AI-assisted tools outperform those relying on traditional methods alone^23,26^. Incorporation of AI models into the decision-making processes for diagnosis and risk stratification seems to be the best current approach for integrating AI in clinical practice; this method balances the strengths of ML with the clinician’s ultimate responsibility for decision-making and positions AI as a supportive tool, enhancing clinical practice without replacing the clinician’s role.

Currently, most AI research in echocardiography centers on diagnostic applications. Echocardiography already plays a key role in assessing hemodynamic status through measures such as IVC collapsibility, LV dimensions, mitral inflow, and E/e′ ratio, particularly in critical care settings^71^. However, its routine use for monitoring is often limited by the need for trained personnel. AI could help address this by automating key measurements and interpretation, making longitudinal monitoring more accessible in point-of-care and resource-limited settings. Additionally, the potential of AI to contribute to treatment planning and management remains underexplored.

## Conclusion

AI and ML have shown considerable potential in improving the accuracy and efficiency of echocardiography, from automated LVEF measurement to wall motion analysis and detection of valvular disease. These technologies address key challenges such as interobserver variability and time-consuming manual interpretation, offering faster, more consistent results. Nevertheless, the integration of AI into routine clinical practice requires overcoming challenges related to generalizability across diverse patient populations and the need for larger, high-quality datasets. Continued research and collaborative efforts between engineers, clinicians, and researchers will be essential to maximize the impact of AI in echocardiography, improving patient outcomes and expanding access to high-quality cardiovascular care.

## Data Availability

No datasets were generated or analysed during the current study.

## References

[1] Lancellotti, P. et al. The use of echocardiography in acute cardiovascular care: recommendations of the European Association of Cardiovascular Imaging and the Acute Cardiovascular Care Association. *Eur. Heart J. Cardiovasc. Imaging* **16**, 119–146 (2015). 25378470 10.1093/ehjci/jeu210

[2] Bunting, K. V. et al. A practical guide to assess the reproducibility of echocardiographic measurements. *J. Am. Soc. Echocardiogr.* **32**, 1505–1515 (2019). 31653530 10.1016/j.echo.2019.08.015

[3] Kusunose, K. et al. Clinically feasible and accurate view classification of echocardiographic images using deep learning. *Biomolecules* **10**, 665 (2020). 32344829 10.3390/biom10050665 PMC7277840

[4] Madani, A., Arnaout, R., Mofrad, M. & Arnaout, R. Fast and accurate view classification of echocardiograms using deep learning. *NPJ Digit. Med.* **1**, 6 (2018). 30828647 10.1038/s41746-017-0013-1 PMC6395045

[5] Jentzer, J. C., Kashou, A. H. & Murphree, D. H. Clinical applications of artificial intelligence and machine learning in the modern cardiac intensive care unit. *Intell. Based Med.* **7**, 100089 (2023).

[6] Pinto-Coelho, L. How artificial intelligence is shaping medical imaging technology: a survey of innovations and applications. *Bioengineering* **10**, 1435 (2023). 38136026 10.3390/bioengineering10121435 PMC10740686

[7] Hughes, J. W. et al. Deep learning evaluation of biomarkers from echocardiogram videos. *EBioMedicine* **73**, 103613 (2021). 34656880 10.1016/j.ebiom.2021.103613 PMC8524103

[8] Sarker, I. H. Machine learning: algorithms, real-world applications and research directions. *SN Comput. Sci.* **2**, 160 (2021). 33778771 10.1007/s42979-021-00592-x PMC7983091

[9] LeCun, Y., Bengio, Y. & Hinton, G. Deep learning. *Nature* **521**, 436–444 (2015). 26017442 10.1038/nature14539

[10] Berg, E. A. R. et al. Fully automatic estimation of global left ventricular systolic function using deep learning in transoesophageal echocardiography. *Eur. Heart J. Imaging Methods Pract.* **1**, qyad007 (2023). 39044786 10.1093/ehjimp/qyad007 PMC11195714

[11] Sfakianakis, C., Simantiris, G. & Tziritas, G. GUDU: Geometrically-constrained Ultrasound Data augmentation in U-Net for echocardiography semantic segmentation. *Biomed. Signal. Process. Control* **82**, 104557 (2023).

[12] Ronneberger, O., Fischer, P. & Brox, T. U-Net: convolutional networks for biomedical image segmentation. in *Lecture Notes in Computer Science* 234–241 (Springer International Publishing, 2015).

[13] Selvaraju, R. R. et al. Grad-CAM: Visual explanations from deep networks via gradient-based localization. *Int. J. Comput. Vis.* **128**, 336–359 (2020).

[14] Kosaraju, A., Goyal, A., Grigorova, Y. & Makaryus, A. N. *Left Ventricular Ejection Fraction*. (StatPearls Publishing, 2023). 29083812

[15] Knackstedt, C. et al. Fully automated versus standard tracking of left ventricular ejection fraction and longitudinal strain: the FAST-EFs multicenter study. *J. Am. Coll. Cardiol.* **66**, 1456–1466 (2015). 26403342 10.1016/j.jacc.2015.07.052

[16] Naser, J. A. et al. Artificial intelligence-based classification of echocardiographic views. *Eur Heart J. Digit. Health* **5**, 260–269 (2024). 38774376 10.1093/ehjdh/ztae015 PMC11104471

[17] Steffner, K. R. et al. Deep learning for transesophageal echocardiography view classification. *Sci. Rep.* **14**, 11 (2024). 38167849 10.1038/s41598-023-50735-8 PMC10761863

[18] Chen, C. et al. Deep learning for cardiac image segmentation: a review. *Front. Cardiovasc. Med.* **7**, 25 (2020). 32195270 10.3389/fcvm.2020.00025 PMC7066212

[19] Liu, X. et al. Deep learning-based automated left ventricular ejection fraction assessment using 2-D echocardiography. *Am. J. Physiol. Heart Circ. Physiol.* **321**, H390–H399 (2021). 34170197 10.1152/ajpheart.00416.2020

[20] Ouyang, D. et al. Video-based AI for beat-to-beat assessment of cardiac function. *Nature* **580**, 252–256 (2020). 32269341 10.1038/s41586-020-2145-8 PMC8979576

[21] Ghorbani, A. et al. Deep learning interpretation of echocardiograms. *NPJ Digit. Med.* **3**, 10 (2020). 31993508 10.1038/s41746-019-0216-8 PMC6981156

[22] Reddy, C. D., Lopez, L., Ouyang, D., Zou, J. Y. & He, B. Video-based deep learning for automated assessment of left ventricular ejection fraction in pediatric patients. *J. Am. Soc. Echocardiogr.* **36**, 482–489 (2023). 36754100 10.1016/j.echo.2023.01.015

[23] Yamaguchi, N., Kosaka, Y., Haga, A., Sata, M. & Kusunose, K. Artificial intelligence-assisted interpretation of systolic function by echocardiogram. *Open Heart* **10**, e002287 (2023). 37460267 10.1136/openhrt-2023-002287 PMC10357654

[24] Olaisen, S. et al. Automatic measurements of left ventricular volumes and ejection fraction by artificial intelligence: clinical validation in real time and large databases. *Eur. Heart J. Cardiovasc. Imaging* **25**, 383–395 (2024). 37883712 10.1093/ehjci/jead280 PMC11024810

[25] He, B. et al. AI-Enabled assessment of cardiac function and video quality in emergency department point-of-care echocardiograms. *J. Emerg. Med.* **66**, 184–191 (2024). 38369413 10.1016/j.jemermed.2023.02.005

[26] Asch, F. M. et al. Deep learning-based automated echocardiographic quantification of left ventricular ejection fraction: a point-of-care solution. *Circ. Cardiovasc. Imaging* **14**, e012293 (2021). 34126754 10.1161/CIRCIMAGING.120.012293

[27] Gohar, E. et al. Artificial Intelligence (AI) versus POCUS expert: a validation study of three automatic AI-Based, real-time, hemodynamic echocardiographic assessment tools. *J. Clin. Med. Res.* **12**, 1352 (2023). 10.3390/jcm12041352 PMC9959768 36835888

[28] Luong, C. L. et al. Validation of machine learning models for estimation of left ventricular ejection fraction on point-of-care ultrasound: insights on features that impact performance. *Echo Res. Pract.* **11**, 9 (2024). 38539236 10.1186/s44156-024-00043-2 PMC10976698

[29] 2023 ACC Expert Consensus on Management of HFpEF: Key Points. A*merican College of Cardiology* https://www.acc.org/Latest-in-Cardiology/ten-points-to-remember/2023/04/17/16/40/2023-acc-expert-consensus-on-hfpef.

[30] Tsao, C. W. et al. Heart disease and stroke statistics-2022 update: a report from the American Heart Association. *Circulation* **145**, e153–e639 (2022). 35078371 10.1161/CIR.0000000000001052

[31] Ho, J. E., Redfield, M. M., Lewis, G. D., Paulus, W. J. & Lam, C. S. P. Deliberating the diagnostic dilemma of heart failure with preserved ejection fraction. *Circulation* **142**, 1770–1780 (2020). 33136513 10.1161/CIRCULATIONAHA.119.041818 PMC7805557

[32] Redfield, M. M. & Borlaug, B. A. Heart failure with preserved ejection fraction: a review. *JAMA* **329**, 827–838 (2023). 36917048 10.1001/jama.2023.2020

[33] Chen, X. et al. Artificial intelligence-assisted left ventricular diastolic function assessment and grading: multiview versus single view. *J. Am. Soc. Echocardiogr.* **36**, 1064–1078 (2023). 37437669 10.1016/j.echo.2023.07.001

[34] Chiou, Y.-A., Hung, C.-L. & Lin, S.-F. AI-Assisted echocardiographic prescreening of heart failure with preserved ejection fraction on the basis of intrabeat dynamics. *JACC Cardiovasc. Imaging* **14**, 2091–2104 (2021). 34147456 10.1016/j.jcmg.2021.05.005

[35] Akerman, A. P. et al. Automated echocardiographic detection of heart failure with preserved ejection fraction using artificial intelligence. *JACC: Adv.* **2**, 100452 (2023). 38939447 10.1016/j.jacadv.2023.100452 PMC11198161

[36] Kobayashi, M. et al. Machine learning-derived echocardiographic phenotypes predict heart failure incidence in asymptomatic individuals. *JACC Cardiovasc. Imaging* **15**, 193–208 (2022). 34538625 10.1016/j.jcmg.2021.07.004

[37] Carluccio, E. et al. Left atrial strain in the assessment of diastolic function in heart failure: a machine learning approach. *Circ. Cardiovasc. Imaging* **16**, e014605 (2023). 36752112 10.1161/CIRCIMAGING.122.014605

[38] Gruca, M. M. et al. Noninvasive assessment of left ventricular end-diastolic pressure using machine learning-derived phasic left atrial strain. *Eur. Heart J. Cardiovasc. Imaging* **25**, 18–26 (2023). 37708373 10.1093/ehjci/jead231

[39] Xu, B. et al. Artificial intelligence machine learning based evaluation of elevated left ventricular end-diastolic pressure: a cleveland Clinic cohort study. *Cardiovasc. Diagn. Ther.* **14**, 788–797 (2024). 39513146 10.21037/cdt-24-128 PMC11538842

[40] Fang, M. Z. et al. Abstract 12374: machine learning-based evaluation of elevated left ventricular end-diastolic pressure. *Circulation* **148**, A12374–A12374 (2023).

[41] Konstam, M. A. et al. Evaluation and management of right-sided heart failure: a scientific statement from the American Heart Association. *Circulation* **137**, e578–e622 (2018). 29650544 10.1161/CIR.0000000000000560

[42] Tokodi, M. et al. Deep learning-based prediction of right ventricular ejection fraction using 2D echocardiograms. *JACC Cardiovasc. Imaging* **16**, 1005–1018 (2023). 37178072 10.1016/j.jcmg.2023.02.017

[43] Kampaktsis, P. N. et al. An attention-based deep learning method for right ventricular quantification using 2D echocardiography: Feasibility and accuracy. *Echocardiography* **41**, e15719 (2024). 38126261 10.1111/echo.15719

[44] Shad, R. et al. Predicting post-operative right ventricular failure using video-based deep learning. *Nat. Commun.* **12**, 5192 (2021). 34465780 10.1038/s41467-021-25503-9 PMC8408163

[45] Liu, S. et al. Artificial intelligence-based assessment of indices of right ventricular function. *J. Cardiothorac. Vasc. Anesth.* **34**, 2698–2702 (2020). 32165105 10.1053/j.jvca.2020.01.024

[46] Holste, G. et al. Severe aortic stenosis detection by deep learning applied to echocardiography. *Eur. Heart J.* **44**, 4592–4604 (2023). 37611002 10.1093/eurheartj/ehad456 PMC11004929

[47] Dai, W., Nazzari, H., Namasivayam, M., Hung, J. & Stultz, C. M. Identifying aortic stenosis with a single parasternal long-axis video using deep learning. *J. Am. Soc. Echocardiogr.* **36**, 116–118 (2023). 36323367 10.1016/j.echo.2022.10.014

[48] Thalappillil, R. et al. Artificial intelligence for the measurement of the aortic valve annulus. *J. Cardiothorac. Vasc. Anesth.* **34**, 65–71 (2020). 31351874 10.1053/j.jvca.2019.06.017

[49] Krishna, H. et al. Fully automated artificial intelligence assessment of aortic stenosis by echocardiography. *J. Am. Soc. Echocardiogr.* **36**, 769–777 (2023). 36958708 10.1016/j.echo.2023.03.008

[50] Wessler, B. S. et al. Automated detection of aortic stenosis using machine learning. *J. Am. Soc. Echocardiogr.* **36**, 411–420 (2023). 36641103 10.1016/j.echo.2023.01.006 PMC10653158

[51] Lachmann, M. et al. Subphenotyping of patients with aortic stenosis by unsupervised agglomerative clustering of echocardiographic and hemodynamic data. *JACC Cardiovasc. Interv.* **14**, 2127–2140 (2021). 34620391 10.1016/j.jcin.2021.08.034

[52] Sánchez-Puente, A. et al. Machine learning to optimize the echocardiographic follow-up of aortic stenosis. *JACC Cardiovasc. Imaging* **16**, 733–744 (2023). 36881417 10.1016/j.jcmg.2022.12.008

[53] Strange, G., Stewart, S., Watts, A. & Playford, D. Enhanced detection of severe aortic stenosis via artificial intelligence: a clinical cohort study. *Open Heart* **10**, e002265 (2023). 37491129 10.1136/openhrt-2023-002265 PMC10373677

[54] Hausleiter, J. et al. Artificial intelligence-derived risk score for mortality in secondary mitral regurgitation treated by transcatheter edge-to-edge repair: the EuroSMR risk score. *Eur. Heart J.* **45**, 922–936 (2024). 38243773 10.1093/eurheartj/ehad871

[55] Sadeghpour, A. et al. An automated machine learning-based quantitative multiparametric approach for mitral regurgitation severity grading. *JACC Cardiovasc. Imaging*10.1016/j.jcmg.2024.06.011 (2024). 39152959 10.1016/j.jcmg.2024.06.011

[56] Bernard, J. et al. Integrating echocardiography parameters with explainable artificial intelligence for data-driven clustering of primary mitral regurgitation phenotypes. *JACC Cardiovasc. Imaging* **16**, 1253–1267 (2023). 37178071 10.1016/j.jcmg.2023.02.016

[57] Yang, F. et al. Automated analysis of doppler echocardiographic videos as a screening tool for valvular heart diseases. *JACC Cardiovasc. Imaging* **15**, 551–563 (2022). 34801459 10.1016/j.jcmg.2021.08.015

[58] Xu, B. & Sanchez-Nadales, A. Artificial intelligence in echocardiographic evaluation of mitral regurgitation: envisioning the future. *JACC Cardiovasc. Imaging*10.1016/j.jcmg.2024.05.026 (2024). 39152963 10.1016/j.jcmg.2024.05.026

[59] Andreassen, B. S., Veronesi, F., Gerard, O., Solberg, A. H. S. & Samset, E. Mitral annulus segmentation using deep learning in 3-D transesophageal echocardiography. *IEEE J. Biomed. Health Inform.* **24**, 994–1003 (2020). 31831455 10.1109/JBHI.2019.2959430

[60] Edwards, L. A. et al. Machine learning for pediatric echocardiographic mitral regurgitation detection. *J. Am. Soc. Echocardiogr.* **36**, 96–104.e4 (2023). 36191670 10.1016/j.echo.2022.09.017

[61] Brown, K. et al. Using artificial intelligence for rheumatic heart disease detection by echocardiography: focus on mitral regurgitation. *J. Am. Heart Assoc.* **13**, e031257 (2024). 38226515 10.1161/JAHA.123.031257 PMC10926790

[62] Martins, J. F. B. S. et al. Towards automatic diagnosis of rheumatic heart disease on echocardiographic exams through video-based deep learning. *J. Am. Med. Inform. Assoc.* **28**, 1834–1842 (2021). 34279636 10.1093/jamia/ocab061 PMC8363807

[63] Peck, D. et al. The use of Artificial Intelligence Guidance for rheumatic heart disease screening by novices. *J. Am. Soc. Echocardiogr.* **36**, 724–732 (2023). 36906047 10.1016/j.echo.2023.03.001

[64] Trenkwalder, T. et al. Machine learning identifies pathophysiologically and prognostically informative phenotypes among patients with mitral regurgitation undergoing transcatheter edge-to-edge repair. *Eur. Heart J. Cardiovasc. Imaging* **24**, 574–587 (2023). 36735333 10.1093/ehjci/jead013

[65] Liu, B. et al. A deep learning framework assisted echocardiography with diagnosis, lesion localization, phenogrouping heterogeneous disease, and anomaly detection. *Sci. Rep.* **13**, 3 (2023). 36593284 10.1038/s41598-022-27211-w PMC9807607

[66] Duffy, G. et al. High-throughput precision phenotyping of left ventricular hypertrophy with cardiovascular deep learning. *JAMA Cardiol.* **7**, 386–395 (2022). 35195663 10.1001/jamacardio.2021.6059 PMC9008505

[67] Goto, S. et al. Artificial intelligence-enabled fully automated detection of cardiac amyloidosis using electrocardiograms and echocardiograms. *Nat. Commun.* **12**, 2726 (2021). 33976142 10.1038/s41467-021-22877-8 PMC8113484

[68] Hussain, K. & Shetty, M. Cardiac sarcoidosis. in *StatPearls* (StatPearls Publishing, 2024). 35201720

[69] Katsushika, S. et al. Deep learning algorithm to detect cardiac sarcoidosis from echocardiographic movies. *Circ. J.* **86**, 87–95 (2021). 34176867 10.1253/circj.CJ-21-0265

[70] Eche, T., Schwartz, L. H., Mokrane, F.-Z. & Dercle, L. Toward generalizability in the deployment of artificial intelligence in radiology: role of computation stress testing to overcome underspecification. *Radiol. Artif. Intell.* **3**, e210097 (2021). 34870222 10.1148/ryai.2021210097 PMC8637230

[71] Porter, T. R. et al. Guidelines for the use of echocardiography as a monitor for therapeutic intervention in adults: a report from the American Society of Echocardiography. *J. Am. Soc. Echocardiogr.* **28**, 40–56 (2015). 25559474 10.1016/j.echo.2014.09.009

